# Supplementary material for: Comprehensive intervention for reducing stigma of autism spectrum disorders: Incorporating the experience of simulated autistic perception and social contact
Source: PLoS One. 2023 Aug 2;18(8):e0288586. doi: 10.1371/journal.pone.0288586 (PMC10395970; doi:10.1371/journal.pone.0288586)
Supplement: S1 Protocol — dx.doi.org/10.17504/protocols.io.q26g7y9e9gwz/v1. (DOCX) [file pone.0288586.s001.docx]

**S1 Protocol. A detailed protocol for this research.** dx.doi.org/10.17504/protocols.io.q26g7y9e9gwz/v1
