# Supplementary material for: Comprehensive intervention for reducing stigma of autism spectrum disorders: Incorporating the experience of simulated autistic perception and social contact
Source: PLoS One. 2023 Aug 2;18(8):e0288586. doi: 10.1371/journal.pone.0288586 (PMC10395970; doi:10.1371/journal.pone.0288586)
Supplement: S1 Dataset — dx.doi.org/10.17605/OSF.IO/QZE39. (DOCX) [file pone.0288586.s002.docx]

**S1 Dataset. Dataset used in the analysis.** dx.doi.org/10.17605/OSF.IO/QZE39
