# Supplementary material for: Comprehensive intervention for reducing stigma of autism spectrum disorders: Incorporating the experience of simulated autistic perception and social contact
Source: PLoS One. 2023 Aug 2;18(8):e0288586. doi: 10.1371/journal.pone.0288586 (PMC10395970; doi:10.1371/journal.pone.0288586)
Supplement: Attachment — Submitted filename: pd.docx [file pone.0288586.s003.docx]

**Abstract**

Described the sample selection, procedure, age, and gender base characteristics of the sample briefly. Introduction is good and well write, need some advance researches.

**Method**

2) Sentence no 134, need rephrasing.

3) Explain the standard procedure and protocol, while conducting experimental research.

4) Result discrepancy while assessing the attitude online (pre-test), while the other two-assessment method face to face with individual. Justify?

5) Participants demographic characteristics such as age, education, financial status, and occupation are missing.

6) How the extraneous variables are controlled while conducting experimental method.

7) Need further tabulation on the bases of participant’s characteristics.

8) Need further analysis clarify the result?

9) Overall manuscript are not according the standard format APA 7^th^
