# Supplementary material for: Comprehensive intervention for reducing stigma of autism spectrum disorders: Incorporating the experience of simulated autistic perception and social contact
Source: PLoS One. 2023 Aug 2;18(8):e0288586. doi: 10.1371/journal.pone.0288586 (PMC10395970; doi:10.1371/journal.pone.0288586)
Supplement: Attachment — Submitted filename: Response to Reviewers.docx [file pone.0288586.s004.docx]

Review Comments to the Author

Reviewer #1: some procedural and statistical errors were found in research paper, the author need special attention to correct these procedural and statistical errors. the method and result section need further clarity and advancement

We described detailed procedure on protocols.io, and conducted additional analysis to confirm that the extraneous variables are controlled in this study. We also added a new table to display participants’ characteristics.

**Abstract**

Described the sample selection, procedure, age, and gender base characteristics of the sample briefly.

We added information of the sample and procedure.

Introduction is good and well write, need some advance researches.

We replaced some references in Introduction with more recent one.

**Method**

2) Sentence no 134, need rephrasing.

As you mentioned, the first sentence in the section of Research design was unnecessary and helpless to understand the research design. We therefore deleted it.

3) Explain the standard procedure and protocol, while conducting experimental research.

We described the protocol of this intervention on protocol.io according to recommendation of the editor. We added supporting information section to the manuscript to inform readers about it.

4) Result discrepancy while assessing the attitude online (pre-test), while the other two-assessment method face to face with individual. Justify?

The follow-up test was not face to face with individual but was online as well as the pretest. We therefore concluded that decrease of the negative affect score and increase of the behavior score at the follow-up test were not caused by the difference between online or face to face. Rather, it is possible that the difference of assessing method might induce increase of the calm score at the workshop period test. We added the explanation of the possibility at line 159.

5) Participants demographic characteristics such as age, education, financial status, and occupation are missing.

Participants’ age was described at line 245 and Table 1.

We could not include questions about education, financial status, and occupation in the sociodemographic questionnaire for a careless mistake. We added the explanation at line 280.

6) How the extraneous variables are controlled while conducting experimental method.

As for allocation bias, we randomly assigned two adjacent days to either the beginning or end groups. It seems that there was no allocation bias in this study, considering the results that there was no difference of age, gender, and interaction with individuals with ASD between the beginning and end groups. We also conducted additional analysis to confirm the allocation bias was controlled (see question 8). As for sampling bias, we could not sufficiently control sampling because we recruited participants through a developmental disability support company and as a results most of them were caregivers or families of individuals with ASD. We discussed this sampling bias in the Limitation section starting from line 570.

7) Need further tabulation on the bases of participant’s characteristics.

We added table of sociodemographic variables as Table 1.

8) Need further analysis clarify the result?

In order to confirm that the allocation bias did not affect the long-term attitude changes, we additionally conducted a two way mixed ANOVA with two tests (within-subject factor; pretest, follow-up test) and two groups (between-subject factor; beginning group, end group).

9) Overall manuscript are not according the standard format APA 7^th^

We checked our manuscript and corrected the style and format, according to PLOS ONE submission Guidelines (<https://journals.plos.org/plosone/s/submission-guidelines>).

Reviewer #2: This is an interesting study and the authors have collected a unique dataset using the cutting-edge methodology. The paper is generally well structured. However, in my opinion, the paper has some shortcomings in regard to some text. In the manuscript, numerous text is vague and rambling (e.g. line 53-57, 61-64, etc.) It is suggested to edit the manuscript carefully and rewrite the long sentences to make them more impactful and easy for the reader.
In discussion, it is suggested to cite literature which is supporting your argument even if the hypothesis is not accepted. In the Limitation section, the author has given recommendations. It is suggested to write recommendations under different headings instead of rambling limitations. Furthermore, there is no need of mentioning results in this section. With Given these shortcomings, the manuscript requires revisions.

We agree that the manuscript was vague and rambling, therefore we rewrite it to be clarify and easy to read. In Introduction, we deleted redundant explanations of references and combined some paragraphs. In discussion, we added a paragraph with citations of related literatures to support our argument at line 556-567. We removed recommendations from the Limitations section to the new section named Future Directions and Conclusion. We also deleted mentions of results. Additionally, we deleted a paragraph mentioning internalized stigma in Limitations section because it is extraneous.
